# Whole-Brain Mapping of the Inputs and Outputs of the Medial Part of the Olfactory Tubercle

**DOI:** 10.3389/fncir.2017.00052

**Published:** 2017-07-28

**Authors:** Zhijian Zhang, Hongruo Zhang, Pengjie Wen, Xutao Zhu, Li Wang, Qing Liu, Jie Wang, Xiaobin He, Huadong Wang, Fuqiang Xu

**Affiliations:** ^1^College of Life Science and Technology, Huazhong University of Science and Technology Wuhan, China; ^2^Center for Brain Science, Key Laboratory of Magnetic Resonance in Biological Systems and State Key Laboratory of Magnetic Resonance and Atomic and Molecular Physics, Wuhan Institute of Physics and Mathematics, CAS Center for Excellence in Brain Science and Intelligence Technology, Chinese Academy of Sciences Wuhan, China; ^3^Wuhan National Laboratory for Optoelectronics Wuhan, China; ^4^College of Life Sciences, Wuhan University Wuhan, China; ^5^Center for Excellence in Brain Science and Intelligence Technology, Chinese Academy of Sciences Shanghai, China

**Keywords:** the medial part of the OT, virus tracing tools, D1R and D2R neurons, neuronal circuitry, whole-brain mapping

## Abstract

The medial part of the olfactory tubercle (OT) is a brain structure located at the interface of the reward and olfactory system. It is closely related to pheromone-rewards, natural reinforcement, addiction and many other behaviors. However, the structure of the anatomic circuitry of the medial part of the OT is still unclear. In the present study, the medial part of the OT was found to be highly connected with a wide range of brain areas with the help of the pseudorabies virus tracing tool. In order to further investigate the detailed connections for specific neurons, another tracing tool – rabies virus was utilized for D1R-cre and D2R-cre mice. The D1R and D2R neurons in the medial part of the OT were both preferentially innervated by the olfactory areas, especially the piriform cortex, and both had similar direct input patterns. With the help of the adeno-associated virus labeling, it was found that the two subpopulations of neurons primarily innervate with the reward related brain regions, with slightly less axons projecting to the olfactory areas. Thus, the whole-brain input and output circuitry structures for specific types of neurons in the medial part of the OT were systematically investigated, and the results revealed many unique connecting features. This work could provide new insights for further study into the physiological functions of the medial part of the OT.

## Introduction

The olfactory tubercle (OT), as its name indicates, is a key part of the olfactory sensory cortex that receives direct inputs from the olfactory bulb (OB) (Pigache, 1970; Xiong and Wesson, 2016). It’s a relatively common brain structure, which is found in many birds and almost all mammals (Wesson and Wilson, 2011). Many specific anatomical features confirm the unique characteristics of the OT over the other brain regions. For example, the OT is the only sensory cortex located in the striatum, and it has many direct or indirect connections with the olfactory system (Xiong and Wesson, 2016). It possesses the “Islands of Calleja” (IC), which is unique among rodents’ brains (Adjei and Wesson, 2015); and dynamic laminations containing “hills” and “valleys,” which are not found in any other cortex structures. These anatomical features also endowed the OT with important roles in behavioral functions. It was reported that the OT was involved in olfactory perception (Zelano et al., 2007; Wesson and Wilson, 2010), odor-guided behaviors (Wesson and Wilson, 2011; Gadziola et al., 2015; Murata et al., 2015), social and sexual responsiveness (Hitt et al., 1973; Agustin-Pavon et al., 2014; DiBenedictis et al., 2015), attention modulation (Zelano et al., 2007), reward seeking and drug related addictive behaviors such as ethanol or cocaine (Ikemoto, 2003, 2005; de Araujo et al., 2009; Gadziola and Wesson, 2016).

Despite this, the OT has a unique anatomy and functional diversity. As the basic anatomical feature and foundation of function, the circuitry structure of the OT has not been thoroughly studied yet. Previous tracing studies which used traditional tracers have revealed that the OT is densely innervated and has efferent projections to most brain areas (DiBenedictis et al., 2015). However, these traditional techniques cannot distinguish synaptic inputs from extra-synaptic innervations or even fibers passing by. Classical dyes have difficulties in revealing the multi-synaptic input pathways. Most importantly, they lack the abilities to label the whole-brain input or output connections for cell-type specific neurons.

The major neuronal compositions of the OT are D1 and D2 dopamine receptor-expressing medium spiny neurons (MSN) (Millhouse and Heimer, 1984; Murata et al., 2015). It has been reported that the D1R and D2R expressing neurons in the OT respond differently to reward and punishment associated odors. The D1R-MSNs in the medial and lateral parts of OT were activated by attractive and aversive associated odors, respectively. However, the D2R-MSNs in the medial part of the OT were activated by aversive associated odors, while the activities of D2R-MSNs in the lateral part of the OT were not changed by sugar-associated odors (Murata et al., 2015). Ethanol-sensitized mice showed reduced D2R but not D1R binding levels in the OT (de Araujo et al., 2009). The D1R-MSN and D2R-MSN of the OT are functionally different, however, their input and output networks have not been studied yet.

The medial part of the OT is involved in pheromonal-reward (DiBenedictis et al., 2015), natural reinforcement (Murata et al., 2015), addiction (Ikemoto, 2003) and many other behaviors (Wesson and Wilson, 2011). Here, the aim was to use a combination of viral toolboxes to reveal the circuitry structure of the medial part of the OT. In this study, the multisynaptic input connections were mapped with the pseudorabies virus (PRV) (Billig et al., 2000). Furthermore, the whole-brain organizations of mono-synaptic inputs to the D1R- or D2R-MSNs in the medial part of the OT were studied, utilizing the modified rabies virus (RV) (Wickersham et al., 2007). At last, the global axonal efferent patterns of these two types of neurons were examined with adeno-associated virus (AAV).

## Materials and Methods

### Animals

All surgical and experimental procedures were conducted in accordance with the guidelines of the Animal Care and Use Committees at the Wuhan Institute of Physics and Mathematics, Chinese Academy of Sciences. Adult male C57BL/6 mice were purchased from Hunan SJA Laboratory Animal Company. The D1R-cre (Drd1a-cre, 262, Gensat) and D2R-cre (Drd2-cre, ER44, Gensat) mice (gifts from Prof. Zhiqi Xiong) used were heterozygote and produced by mating the transgenic male mice with C57BL/6 females. All Animals were housed with their littermates in a dedicated housing room with a 12/12 h light/dark cycle, and food and water were available *ad libitum*.

### Virus Information

The viral tools were all packaged by BrainVTA (BrainVTA Co., Ltd., Wuhan, China) and all aliquots were stored at -80°C.

For the retrograde trans-multisynaptic tracer, the PRV-152, which expresses EGFP driven by CMV promoter (Billig et al., 2000), was tittered at about 1 × 10^9^ infecting unit per milliliter. For rabies viruses, the titer of RV-EnvA-ΔG-dsRed was about 2 × 10^8^ infecting unit per milliliter. For AAV viruses, the AAV9-EF1a-floxed-EGFP, AAV9-EF1a-Dio-GFP-TVA and AAV9-EF1a-DiO-RV-G were all packaged into 2/9 serotype and tittered at about 3 × 10^12^ genome copies per milliliter.

### Stereotactic Surgery

All procedures on animals were performed in Biosafety level 2 (BSL-2) animal facilities as before (Yang H. et al., 2016; Yang Y. et al., 2016). Animals were anesthetized with chloral hydrate (400 mg/kg, i.p.), and placed in a stereotaxic apparatus (Item: 68030, RWD, Shenzhen, China). During surgery and virus injection, all animals were kept anesthetized with isoflurane (1–1.5%). The skull above the targeted areas was thinned with a dental drill and removed carefully. Injections were conducted with a syringe pump (Item: 53311, Quintessential stereotaxic injector, Stoelting, United States) connected to a glass micropipette with a tip diameter of 10–15 μm. The glass micropipette was held for an extra 10 min after the completion of the injection and then slowly retreated. After the surgery, the incisions were stitched and lincomycin hydrochloride and lidocaine hydrochloride gel was applied to prevent inflammation and alleviate pain for the animals.

To retrograde trace the multiple synaptic afferents of the medial part of the OT, the PRV-152 (200 nl) was unilaterally injected into the adult male C57BL/6 mice with the following coordinates: AP, 1.20 mm; ML, 1.10 mm; and DV, -5.50 mm. The mice were perfused at approximately 40 and 64 h after virus injection, respectively.

To retrograde trace the whole-brain mono-synaptic inputs for the D1R- and D2R-MSNs in the medial part of the OT, the mixture of AAV9-EF1a-Dio-GFP-TVA and AAV9-EF1a-DiO-RV-G (volume ratio: 1:1, 100–150 nl in total) was injected into the D1R-cre and D2R-cre mice, respectively. Then, 300 nl RV-EnvA-ΔG-dsRed was injected into the medial part of the OT at the same injection site with the AAV mixture after 2 weeks. One week after the RV injection, the mice were perfused for brain collection.

To identify the whole-brain axonal efferent patterns of D1R- and D2R-MSNs in the medial part of the OT, AAV9-EF1a-floxed-EGFP (150 nl) was injected into the D1R-cre and D2R-cre animals, respectively. Eight weeks after virus injection, the mice were perfused for brain collection.

### Slice Preparation and Confocal Imaging

The mice were anesthetized with chloral hydrate (10% W/V, 500 mg/kg body weight, i.p.), and perfused transcardially with PBS (5 min), followed by ice-cold 4% paraformaldehyde (PFA, 158127 MSDS, sigma) dissolved in PBS (5 min). The brain tissues were carefully removed and post-fixed in PBS containing 4% PFA at 4°C overnight, and then equilibrated in PBS containing 25% sucrose at 4°C for 72 h. The 40 μm thick coronal slices of the whole brain were obtained using the cryostat microtome and stored at -20°C.

For RV or AAV labeled samples, every sixth section of the brain slices were selected, stained with DAPI, washed with PBS, mounted with 90% glycerol (in PBS) and sealed with nail polish.

For PRV-152 labeled samples, the procedures for immunohistochemistry were performed as before (Wei et al., 2015). Every sixth section of the brain slices were selected and stained with GFP (abcam, ab290, 1: 1000) and DAPI, and then mounted and sealed as described above.

All of the images were captured with the TCS SP8 fluorescence laser scanning confocal microscope (Leica, China) or the Olympus VS120 virtual microscopy slide scanning system (Olympus, Shanghai, China).

### Data Analysis

For cell counting, the boundaries of brain regions were delineated manually with Photoshop based on the Allen Brain Atlas. The labeled neurons were quantified semi-automatically using FIJI and the cell counter plugin in ImageJ.

For the fiber intensity quantification, the images were captured with the same parameters. The boundaries of brain regions were delineated manually with ImageJ based on the Allen Brain Atlas. The mean gray value of fibers in different brain regions were then quantified automatically using the “Measure” plugin in ImageJ.

For statistical analyses, student’s *t*-tests, Mann–Whitney *U*-tests were performed to determine statistical differences using SPSS (version 13.0). Statistical significance was set at ^∗∗∗^*P* < 0.001; ^∗∗^*P* < 0.01 and ^∗^*P* < 0.05. All data values were presented as mean ± SEM. Graphs were drawn using SigmaPlot (version 10.0).

## Results

### The Medial Part of the OT Integrates Extensive Anatomical Inputs from Many Brain Regions

The OT is a multisensory region and is highly interconnected with other brain areas. To reveal the input networks, the tracing tool – PRV-152 (Billig et al., 2000), a GFP-expressing retrograde trans-multisynaptic tracer, was utilized to map the whole-brain inputs of the medial part of the OT.

**FIGURE 1:**
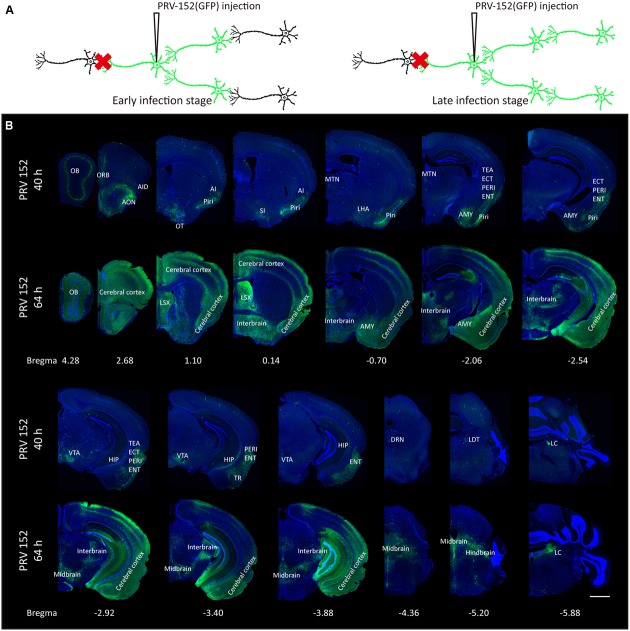
Whole-brain mapping of the medial part of the OT inputs. **(A)** The schematic for PRV-152 retrograde tracing trans-multisynaptic connected neural networks. The PRV labeled circuit stages depends on infection time. **(B)** The whole-brain PRV labeled input network patterns of the medial part of the OT at two different infection times. As the infection course prolonged, the PRV labeled a much wider range of brain regions. The scale bar: 1000 μm.

**FIGURE 2:**
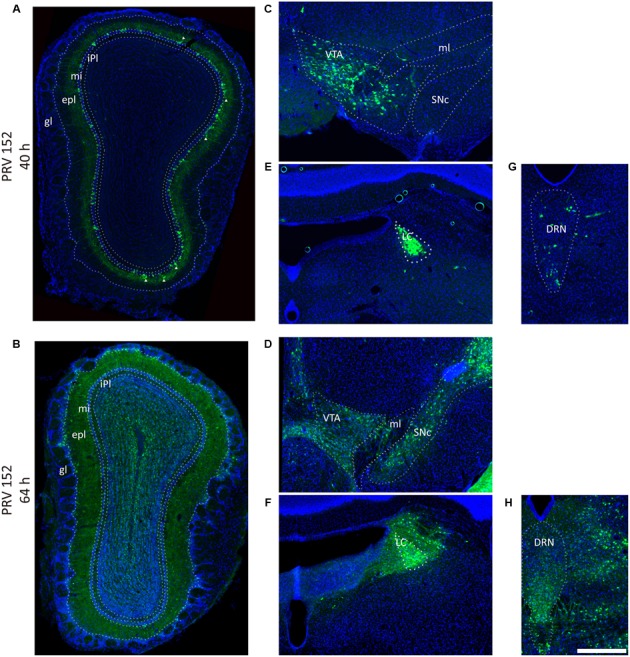
Pseudorabies virus (PRV) labeled the olfaction, reward, and modulation related brain areas. **(A,B)** PRV-152 labeled neurons were mainly distributed in the mitral cell layer, and also sparsely observed in the external plexiform layer (marked by triangle) in the OB after initially infection (40 h) **(A)**, and spread to the entire OB as the infection time prolonged (64 h) **(B)**. **(C–H)** The PRV also labeled neurons in reward-related brain regions including the VTA (**C**, 40 h; **D**, 64 h) and modulation systems like LC (**E**, 40 h; **F**, 64 h) and DRN (**G**, 40 h; **H**, 64 h). In each brain region, the prolonged infection time lead to a wider labeled range. The scale bar: 200 μm.

Since the PRV labeling strategy for input circuitries is time-dependent (**Figure 1A**), the labeled patterns of the whole-brain were examined at two different time points post-infection (**Figures 1B**, **2**). At the earlier infection stage of 40 h, many discrete brain areas were specifically labeled, including both mitral cell layers and external plexiform layers of the OB, anterior olfactory nucleus (AON), orbital cortex (ORB), agranular insular cortex (AI), piriform cortex (Piri), substantia innominata (SI), lateral hypothalamus (LHA), midline group of dorsal thalamus (MTN), amygdala (AMY), temporal association cortex (TEA), entorhinal cortex (ECT), perirhinal cortex (PERI), entorhinal cortex (ENT), hippocampus (HIP), ventral tegmental area (VTA), amygdalopiriform transition area (TR), dorsal raphe nuclei (DRN), laterodorsal tegmental nucleus (LDT) and locus coeruleus (LC), et al. Those initially labeled nuclei were possibly direct connections or the strongest inputs for the medial part of the OT. With a longer infection time (64 h post PRV-152 injection), a much wider range of brain regions were observed with GFP-positive labels, including the entire OB, AMY and lateral septal complex (LSX), most parts of the cerebral cortex, interbrain, midbrain and hindbrain. These results indicated that the medial part of the OT received various direct or indirect neural inputs from the whole brain, suggesting it might play a role in the integration of the information from multiple inputs.

### Direct Input Patterns for D1R and D2R Neurons in the Medial Part of the OT

**FIGURE 3:**
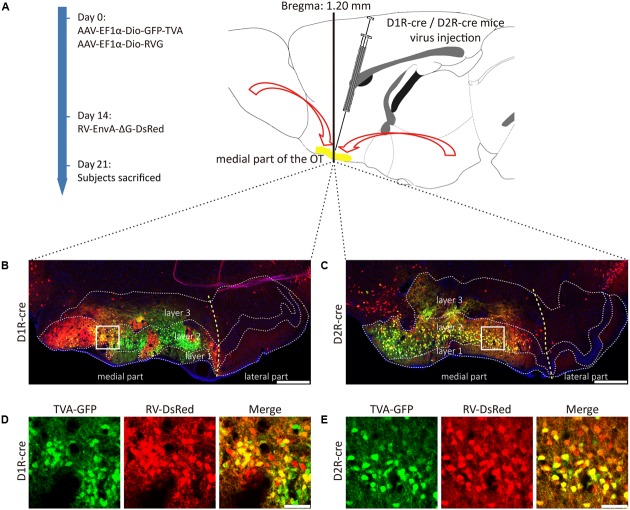
Rabies virus (RV) trans-monosynaptic tracing from the D1R- and D2R-MSNs of the medial part of the OT. **(A)** The schematic for the tracing study. **(B,C)** The representative coronal brain sections near the injection sites. The starter cells indicated by the co-labeling of AAV (GFP) and RV (Red), which were mainly distributed in the medial part of the OT in both types of mouse lines (**B**, D1R-cre mice; **C**, D2R-cre mice). The scale bar: 250 μm. **(D,E)** The magnifications of the starter cells in the medial part of the OT (**D**, D1R-cre mice; **E**, D2R-cre mice). The scale bar: 50 μm.

The PRV tracing approach is a method used for screening out the global input networks, which send information to the medial part of the OT. However, it is difficult to distinguish between weak direct connections and strong indirect ones. Besides, it lacks cell type specificity. The direct input neural connections for D1R- and D2R-MSNs in the medial part of the OT are still unclear. This issue was investigated with the trans-mono-synaptic RV tracing method from D1R- and D2R-MSNs, respectively (**Figure 3A**).

D1R- or D2R-MSNs were labeled with GFP expressed by AAV9-EF1a-Dio-GFP-TVA. The injection site was examined and it was found that the AAV infected neurons were distributed in three layers of the medial part of the OT for these two types of neurons, but extremely abundant in the dense cell layer (layer 2). The yellow staining neurons indicated that the starter cells were initially co-infected by AAV-helper and RV (**Figures 3B–E**). The starter cells were checked carefully to guarantee that their distributions were mostly restricted to the medial part of the OT. Otherwise, the data from these animals should be excluded.

**FIGURE 4:**
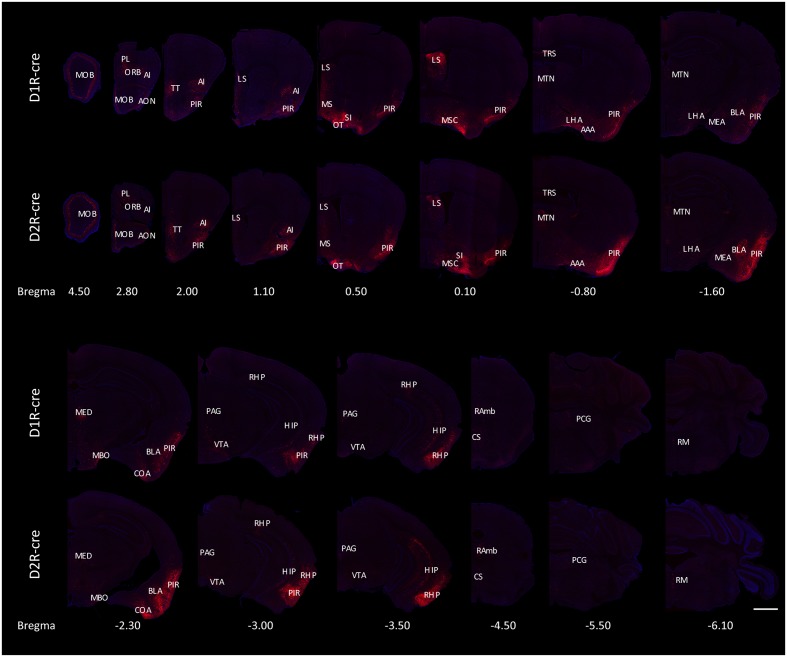
Ipsilateral coronal brain slices series from representative RV-labeled mice showed the whole-brain input patterns of D1R and D2R neurons in the medial part of the OT. A substantial number of input neurons were observed in many dissected brain areas, and with a similar distribution pattern for both D1R-cre and D2R-cre mice. The Scale bar: 1000 μm.

The RV labeled regions (red signals) within several coronal brain slices were displayed from the D1R-cre and D2R-cre mice, respectively (**Figure 4**). A substantial number of input neurons were observed in many discrete nuclei for these two cre-line mice, with a similar distribution pattern.

**FIGURE 5:**
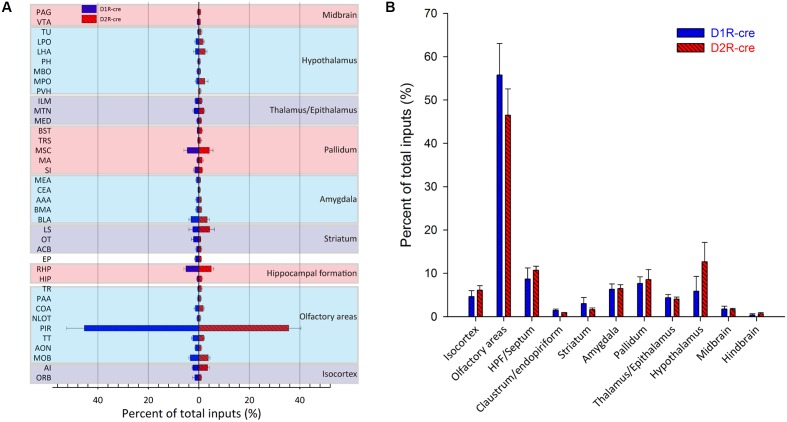
Quantitative analysis of the whole-brain input proportions of D1R and D2R neurons in the medial part of the OT. The ipsilaterally labeled cells by the RV were quantified and the input proportions were normalized by the total ipsilateral input numbers of every mouse. **(A,B)** 38 dissected brain areas **(A)** and 11 brain regions **(B)** with no less than 0.5% average input proportions were selected from eight D1R-cre or D2R-cre mice and displayed here. The inputs to D1R and D2R neurons are labeled in blue and red, respectively. Both subpopulations of neurons were primarily innervated by the olfactory areas, but the input proportions were not significantly different.

In order to check whether the input intensity for each nucleus was different between these two cre-line mice, the numbers of labeled cells within every brain structure were quantified. Since most of the red signals were distributed ipsilaterally, the ipsilateral labeled cells were counted for quantification to compare. The labeled cells for each nucleus were normalized by the total number of input neurons for each animal. Nuclei providing input rates greater than 0.5% were chosen to be analyzed from eight D1R-cre or D2R-cre mice. A total of 38 nuclei and their input proportions were displayed in **Figure 5A**. Despite the functional differences of D1R- and D2R- neurons in the medial part of the OT, it was found that both subpopulations of infected neurons had a similar input distribution pattern within the whole brain. For both D1R-cre and D2R-cre mice, the nuclei of Piri had the highest input proportion (45.43 ± 7.03% for D1R neurons; 35.63 ± 4.84% for D2R neurons) than any other nuclei. The retrohippocampal (RHP) had the second most input neurons (5.12 ± 0.81% for D1R neurons; 4.93 ± 1.04% for D2R neurons) for both mice. The Piri, RHP, MSC, MOB, BLA and TT were the six major presynaptic nuclei that projected to the D1R neurons, whereas, the Piri, RHP, LS, MSC, MOB and AI made up the top six input nuclei for the D2R neurons in the medial part of the OT. Although the rankings of the input proportions were slightly different between these two subpopulations of neurons in the two kinds of mice, the input percentages of each nucleus to D1R and D2R neurons did not show significant difference.

The labeled neurons were further investigated by integration into 11 intact brain regions instead of discrete nuclei (**Figure 5B**). As its name indicates, in both cell types of mice, the medial part of the OT received approximately half of the direct inputs from the olfactory areas (55.77 ± 7.29% for D1R neurons; 46.48 ± 6.08% for D2R neurons). The top three presynaptic input brain regions for D1R and D2R neurons were from olfactory areas, HPF/Septum and pallidum; olfactory areas, hypothalamus and HPF/Septum, respectively. It was noted that even though all of the starter cells expressed dopamine receptors, the midbrain which was enriched with dopaminergic neurons was only weakly labeled (1.79 ± 0.62% for D1R neurons; 1.66 ± 0.28% for D2R neurons) compared with the other brain regions. Despite the different ranks of presynaptic input brain regions for D1R and D2R neurons in the medial part of the OT, the input percentage of each brain region was still not significantly different between these two types of neurons.

**FIGURE 6:**
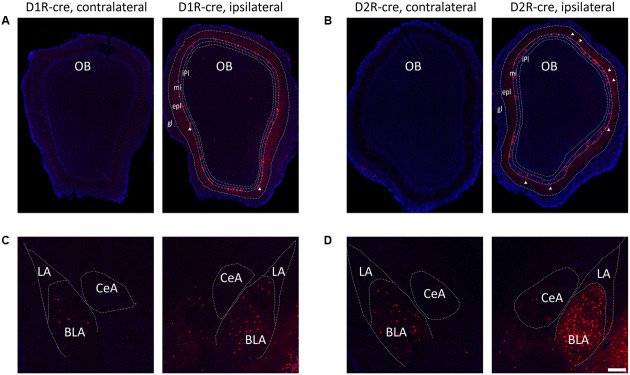
Bilateral input connections of D1R and D2R neurons in the medial part of the OT labeled by the RV. **(A,B)** Both cell types of neurons were mainly connected by ipsilateral brain areas, such as the OB (**A**, D1R-cre mice; **B**, D2R-cre mice; White triangle marked neurons labeled in the external plexiform layer). **(C,D)** However, some contralateral brain areas were still sparsely labeled by RV retrograde tracing. The BLA was densely traced among these weakly labeled contralateral brain areas (**C**, D1R-cre mice; **D**, D2R-cre mice). The Scale bar: 200 μm.

Although the majority of red signals were distributed in the ipsilateral hemisphere, retrograde labeled cells were still sparsely observed in some contralateral brain regions (**Figure 6**). Thus the bilateral input patterns of D1R and D2R neurons in the medial part of the OT were also summarized. The results were presented in **Table 1**. Only several brain areas directly innervate D1R and D2R neurons in the medial part of the OT with a bilateral manner (**Figure 6**). These results indicated that the medial part of the OT mainly processes the neural signals from ipsilateral inputs; however, it could also integrate some information from bilateral side.

**Table 1:** Brain areas formed bilateral inputs or reciprocal connections to the D1R and D2R neurons in the medial part of the OT.

Brain areas	Abbreviations	Bilateral inputs to the medial part of the OT		The axonal projections of the medial part of the OT		Reciprocal connections	
		D1R-cre (*n* = 4)	D2R-cre (*n* = 4)	D1R-cre (*n* = 3)	D2R-cre (*n* = 3)	D1R-cre	D2R-cre
Periaqueductal gray	PAG	+	+			-	-
Ventral tegmental area	VTA	+	+	^∗∗∗∗^	^∗∗^	+	+
Tuberal nucleus	TU	-	-			-	-
Lateral preoptic area	LPO	+	+			-	-
Lateral hypothalamic area	LHA	+	+	^∗∗^	^∗∗^	+	+
Posterior hypothalamic nucleus	PH	+	+			-	-
Mammillary body	MBO	+	+		^∗∗∗^	-	+
Medial preoptic area	MPO	+	+			-	-
Paraventricular hypothalamic nucleus	PVH	-	-			-	-
Intralaminar nuclei of the dorsal thalamus	ILM	-	-			-	-
Midline group of the dorsal thalamus	MTN	+	+			-	-
Medial group of the dorsal thalamus	MED	+	+	^∗∗∗∗^	^∗∗^	+	+
Bed nuclei of the stria terminalis	BST	-	+			-	-
Triangular nucleus of septum	TRS	+	+			-	-
Medial septal complex	MSC	+	+			-	-
Magnocellular nucleus	MA	-	+			-	-
Substantia innominata	SI	+	+			-	-
Medial amygdalar nucleus	MEA	-	-	^∗∗∗∗^	^∗∗∗∗^	+	+
Central amygdalar nucleus	CEA	-	-			-	-
Anterior amygdalar area	AAA	-	+	^∗∗∗∗^	^∗∗^	+	+
Basomedial amygdalar nucleus	BMA	+	+	^∗∗^		+	-
Basolateral amygdalar nucleus	BLA	+	+			-	-
Lateral septal nucleus	LS	+	+			-	-
Olfactory tubercle	OT	+	+			-	-
Nucleus accumbens	ACB	-	-			-	-
Endopiriform nucleus	EP	-	-		^∗^	-	+
Retrohippocampal region	RHP	+	+			-	-
Hippocampal region	HIP	+	+		^∗^	-	+
Postpiriform transition area	TR	-	-			-	-
Piriform-amygdalar area	PAA	+	-			-	-
Cortical amygdalar area	COA	+	+	^∗∗^		+	-
Nucleus of the lateral olfactory tract	NLOT	+	+	^∗∗^		+	-
Piriform area	PIR	+	+	^∗^		+	-
Taenia tecta	TT	+	+	^∗∗∗∗^		+	-
Anterior olfactory nucleus	AON	+	+	^∗∗^	^∗^	+	+
Main olfactory bulb	MOB	-	-	^∗∗^	^∗∗∗^	+	+
Agranular insular area	AI	+	+			-	-
Orbital area	ORB	+	+			-	-
Dorsomedial nucleus of the hypothalamus	DMH	-	+			-	-
Zona incerta	ZI	-	+			-	-
Accessory olfactory bulb	AOB	-	-	^∗^	^∗^	-	-
Epithalamus	EPI	-	-	^∗∗^	^∗^	+	+

*Brain areas marked with “+” means formed bilateral inputs or reciprocal connections to the medial part of the OT; while “-” indicates providing only unidirectional connections or received ipsilateral inputs. For the axonal projections, ^∗^ indicates the mean intensity of fiber distribution: ^∗^1–30; ^∗∗^30–50; ^∗∗∗^50–70; ^∗∗∗∗^70–120; ^∗∗∗∗∗^>120*.

### The Global Axonal Efferent Patterns of D1R and D2R Neurons in the Medial Part of the OT

**FIGURE 7:**
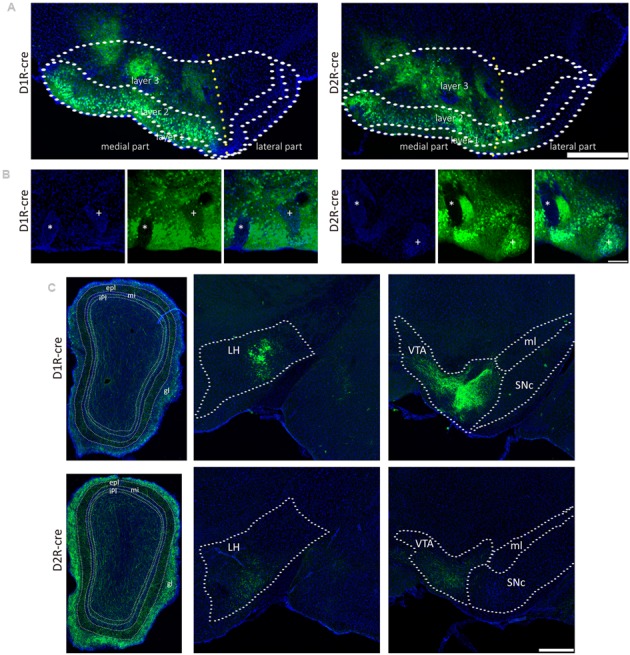
The axonal projections of D1R and D2R neurons in the medial part of the OT. **(A)** The representative coronal brain sections near the injection sites. AAV9-EF1a-floxed-EGFP was injected into the medial part of the OT of either D1R-cre (Left) or D2R-cre mice (Right). The Scale bar: 1000 μm. **(B)** Magnifications of the medial part of the OT infected by AAV injection. The ICs were negatively (^∗^) or weakly (+) stained by AAV in the D1R-cre mice (Left); and were negatively (^∗^) or extensively (+) labeled in the D2R-cre mice (Right). The Scale bar: 250 μm. **(C)** Both subpopulations of the neurons (Top, D1R neurons; Bottom, D2R neurons) send dense axonal projections to many brain areas, including the OB, LH and VTA. The Scale bar: 1000 μm.

The global axonal efferent patterns of D1R and D2R neurons in the medial part of the OT were examined using the AAV labeling approach (**Figure 7A**). The representative figures from the two cre-lines showed both the overviews and magnifications of infected neurons adjacent to the injection sites (**Figures 7A,B**). It was observed that the Islands of Calleja (ICs) were diversely stained by AAV. The D1R-expressing neurons were either undetected or weakly distributed in ICs. However, ICs might contain either negative or extensive D2R-expressing neurons (**Figure 7B**). These results showed that the ICs within the medial part of the OT might possess different neurochemical features from each other, indicating that an individual IC might be functionally heterogeneous. In contrast to any other olfactory sensory cortex that innervates the contralateral brain areas through anterior commissure (Aco) (Cummings et al., 1997; Ito et al., 2010), both types of neurons in the medial part of the OT sent very few contralateral projections, and none of them passed through Aco. Both neural subpopulations had the strongest projections to the pallidum. The hypothalamus and AMY also received many projections. Unlike the input patterns, the olfactory areas received weaker fibers from D1R and D2R neurons in the medial part of the OT. Within the olfactory areas, the accessory olfactory bulbs (AOB) also received projections from both types of neurons (**Figure 7C** and **Table 1**). It was also interesting to find that the VTA and SNc in the midbrain received projections from D1R and D2R neurons in the medial part of the OT, which were stronger from the D1R neurons (**Figure 7C** and **Table 1**). Together with the input patterns, both D1R and D2R neurons in the medial part of the OT formed lots of reciprocal connections within the brain, which is presented in the **Table 1**.

Since both D1R- and D2R-MSNs are GABAergic (Kupchik et al., 2015), the results of the study indicated that the medial part of the OT was able to form a wide range of inhibitory efferent connections within the whole brain. These inhibitory efferent mainly targeted the reward-related brain regions including the pallidum, hypothalamus, amygdale and midbrain; and lower levels of efferent arrived at the olfactory areas including the OB, AOB, and Piri.

## Discussion

The OT is a brain structure with many unique features, both in anatomy and function (Wesson and Wilson, 2011; Xiong and Wesson, 2016). The traditional tract-tracing studies have provided overviews of the input and output patterns of the OT. However, these circuitry connection studies have never been extended to different cell types. In the present study, these issues were studied focusing on the medial part of the OT. The results indicated that the medial part of the OT was indeed highly connected with many areas in the whole brain by a wide range of nuclei, either directly or indirectly. Besides, D1R and D2R expressing neurons in the medial part of the OT received similar direct inputs. They were most preferentially innervated by the olfactory areas, especially in the Piri. Furthermore, both neural subpopulations primarily send projections to the reward-related brain regions including pallidum, hypothalamus, amygdale and midbrain; while the olfactory areas received only relatively weaker axonal innervations from both types of neurons. This study systematically investigated the whole-brain input and output circuitry structures of cell-type specific neurons within the medial part of the OT.

### Input Patterns of the Medial Part of the OT

Previous studies have proposed that the OT was a highly interconnected brain region (Wesson and Wilson, 2011). In the present study, the results confirmed that the circuitry structure of the medial part of the OT were comparably complicated. Both the RV tracing and the earlier PRV infection tracing results revealed that the medial part of the OT was directly innervated by numerous discrete brain areas, especially the olfactory areas. In both studies (**Figures 2A**, **6A,B**), although the majority of virus infected somas were found in the mitral cell layer, the minority of infected cells were also observed in the external plexiform layer, indicating that the OT is likely to be directly innervated by both mitral cells and tufted cells. This result is also consistence with Igarashi’s study by single-cell labeling with BDA (Igarashi et al., 2012). They found that both mitral cells and tufted cells sent axon collaterals to the OT. The PRV prolonged infection data further suggested that a much wider range of brain regions indirectly connects the medial part of the OT. These input network features underline the potential abilities of the medial part of the OT to collaborate multiple information inputs.

Rabies virus tracing results revealed similar direct input patterns of D1R and D2R neurons in the medial part of the OT. The two subpopulations of neurons showed different activity patterns in certain behaviors (Gadziola and Wesson, 2016). Besides this, the biochemical features and neural plasticity are different between the D1R- and D2R-MSN in the striatum (de Araujo et al., 2009; Gerfen and Surmeier, 2011; Keeler et al., 2014). Thus, whether these two types of neurons in the medial part of the OT are connected by different cell subpopulations/types within the similar upstream regions still remains to be clarified. Further studies using double tracing from D1R and D2R neurons within the same animal, and immunochemical staining for different cell types of the input neurons are needed to solve this issue. Although RV tracing was derived from dopamine receptor expressing neurons, the midbrain, a brain region abundant with dopaminergic neurons, was just weakly labeled. This result is similar to the previous studies in the dorsal striatum (Wall et al., 2013; Deng et al., 2015). Considering the fact that the striatum, including the medial part of the OT, is intensively projected by dopaminergic neurons, the weak mono-synaptic RV labeling was proposed to be the result of extra-synaptic modulation (Groves et al., 1994; Descarries et al., 1996; Hanley and Bolam, 1997) or specialized synaptic structures between dopaminergic neurons and the MSNs (Wall et al., 2013). Together with previous studies, our results indicated that dopaminergic neurons are likely to innervate the striatum MSNs with a uniform synaptic mechanism.

We found that the numbers of both starter cells and input neurons labeled by rabies seems to be higher in the D2R-cre mice than the D1R-cre ones (data not shown). It was reported that the D2R+ neurons also contain cholinergic interneurons (Bertran-Gonzalez et al., 2008). Thus, the higher numbers of starter cells and inputs for D2R-cre mice could be due to the labeling of these additional mixed populations.

### The Axonal Projecting Patterns of the Medial Part of the OT

In contrast with previous studies that reported the OT provided few association fibers (Haberly and Price, 1978), our results demonstrated that both D1R and D2R neurons in the medial part of the OT had extensive efferent projections, which is consistent with other different studies (Ikemoto, 2007; Wesson and Wilson, 2011). The medial part of the OT possessed many unique projection features.

Although the medial part of the OT is mainly innervated by the olfactory areas, its major efferent targets are reward-related brain areas. Besides, it was shown that both the D1R and D2R neurons in the medial part of the OT projected to the midbrain, which has never been reported before. The traditional tract-tracing methods lack cell-type specificity and are easily attenuated along the distant and fine axonal fibers. The results that the D2R neurons project to the midbrain is quite different from the widely known direct and indirect pathways within the dorsal stratum (Keeler et al., 2014) and the NAc (Kupchik et al., 2015). These results could provide a new insight for the classical striatal direct and indirect pathways for D1R and D2R neurons.

Unlike most of the olfactory systems, the medial part of the OT did not contralaterally innervate brain areas through the Aco. Instead, it mainly provided ipsilateral projections, which is very different from previous reports (Shafa and Meisami, 1977). Although the axonal distributions in the olfactory areas were not the most dominating, they were still very dense. Fibers from D1R and D2R neurons in the medial part of the OT were both observed in different layers of the entire OB. Given that both D1R and D2R neurons were GABAergic (Kupchik et al., 2015), the medial part of the OT seemed to be able to provide inhibitory feedback projections to the OB. It’s considered that the majority of centrifugal feedback arising in the olfactory cortex projected to the OB was excitatory (Shipley and Ennis, 1996; Balu et al., 2007; Gao and Strowbridge, 2009; Su et al., 2009). Thus, the medial part of the OT might regulate the OB in a different feedback mechanism from the classical olfactory cortex. It was found that the AOB was also projected from the D1R and D2R neurons in the medial part of the OT. Since the medial part of the OT is also highly afferent by and efferent to the OB, these results suggested a role for the medial part of the OT in coordinating signal processing between the OB and the AOB.

The present study used the AAV tracing approach to reveal a lot of unique projection features from the medial part of the OT. Whether these axon fibers were indeed synaptically connected or just passed by the targeted brain areas; and whether the entire OT possesses a uniform output network, are still unclear. Further studies using anterograde trans-synaptic tracing tools, such as the H129 strain of herpes simplex virus, would be helpful to investigate this hypothesis.

Although the present work revealed the input and output neural circuitries of the medial part of the OT with different viral tools, it should be noticed that the border for the medial part of the OT could be potentially imprecisely divided. A precise definition for the medial-lateral division should depend on discrepant expressions of molecular markers or differential cellular morphologies with different functional roles between the two regions, which had rarely been reported as far as we know by now. However, it’s not easy to specifically infect the entire long and narrow tubercle while leave the adjacent brain regions, such as the ventral pallidum, uninfected by viral injecting. To both follow the previous works (Ikemoto, 2003; DiBenedictis et al., 2015; Murata et al., 2015) and lower the possibility of unspecific infections, we aimed to study only the medial part of the OT. Finally, the border for the medial part of the OT was divided at the most gyrated part of the layer 2, and containing the superficially located Islands of Calleja and surrounded cortex-like compartments according to the limited references (Murata et al., 2015; Xiong and Wesson, 2016). Besides, it’s also a difficult problem to perfectly demark the OT from the ventral pallidum. In the present study, the layers of the OT were also divided by reference to Xiong’s work (Xiong and Wesson, 2016). The border of the layer 3 was demarked by the deep layer’s islands of Calleja.

In summary, the input and output circuitry structures at the level of specific cell types within the medial part of the OT were investigated. The findings revealed many unique features of circuitry connections for the medial part of the OT, and could provide new insights for further study into the physiological functions of it.

## Author Contributions

ZZ and FX developed the idea, ZZ, HZ, PW, XZ, and LW performed the experiments, ZZ, QL, XH, JW, HW, and FX analyzed the data, ZZ, QL, JW, and FX conceived the manuscript and wrote the text, and ZZ generated the figures.

## Conflict of Interest Statement

The authors declare that the research was conducted in the absence of any commercial or financial relationships that could be construed as a potential conflict of interest.
